# Phytochemical Analysis and Anti-Cancer Properties of Extracts of *Centaurea castriferrei* Borbás & Waisb Genus of *Centaurea* L

**DOI:** 10.3390/molecules27217537

**Published:** 2022-11-03

**Authors:** Joanna Kubik, Łukasz Waszak, Grzegorz Adamczuk, Ewelina Humeniuk, Magdalena Iwan, Kamila Adamczuk, Mariola Michalczuk, Agnieszka Korga-Plewko, Aleksandra Józefczyk

**Affiliations:** 1 Independent Medical Biology Unit, Faculty of Pharmacy, Medical University of Lublin, 8b Jaczewski Street, 20-093 Lublin, Poland; 2 Department of Pharmacognosy with Medicinal Plant Laboratory, Faculty of Pharmacy, Medical University of Lublin, 1 Chodzki Street, 20-093 Lublin, Poland; 3 Department of Toxicology, Faculty of Pharmacy, Medical University of Lublin, 8 Chodzki Street, 20-093 Lublin, Poland; 4 Department of Biochemistry and Molecular Biology, Faculty of Medical Sciences, Medical University of Lublin, 1 Chodzki Street, 20-093 Lublin, Poland

**Keywords:** centaurea, *Centaurea castriferrei* Borbás & Waisb, Asteraceae, phenolic content, flavonoid, antioxidant potential, anticancer activity

## Abstract

The *Centaurea* L. (Asteraceae) genus includes many plant species with therapeutic properties. *Centaurea castriferrei* Borbás & Waisb is one of the least known and least described plants of this genus. The aim of the study was the phytochemical analysis of water and methanol–water extracts (7:3 *v*/*v*) obtained from the aerial parts of the plant as well as evaluation of their anticancer activity. Quantitative determinations of phenolic compounds and flavonoids were performed, and the antioxidant potential was measured using the CUPRAC method. The RP-HPLC/DAD analysis and HPLC-ESI-QTOF-MS mass spectroscopy were performed, to determine the extracts’ composition. The antiproliferative activity of the obtained extracts was tested in thirteen cancer cell lines and normal skin fibroblasts using MTT test. Regardless of the extraction method and the extractant used, similar cytotoxicity of the extracts on most cancer cell lines was observed. However, the methanol–water extracts (7:3 *v*/*v*) contained significantly more phenolic compounds and flavonoids as well as showing stronger antioxidant properties in comparison to water extracts. *Centaurea castriferrei* Borbás & Waisb is a rich source of apigenin and its derivatives. In all tested extracts, chlorogenic acid and centaurein were also identified. In vitro research revealed that this plant may be a potential source of compounds with anticancer activity.

## 1. Introduction

For centuries, plants have been used both in the treatment and prevention of diseases, as they contain a large amount of compounds with a broad spectrum of biological activity. The constant interest in acquiring new plant-based compounds that can support the treatment of many diseases leads to the dynamic development in the field of research of compounds obtained from plant material [1,2]. Plant extracts are used as anti-inflammatory and antimicrobial agents, and numerous studies have been carried out in terms of the development and identification of new anti-cancer agents, which have been repeatedly confirmed in studies [3]. Currently, there is a lot of research carried out around the world in order to identify new plant species with anti-cancer activity that are an alternative to toxic chemotherapeutic agents [4,5].

Due to the progressive resistance of cancer cells to applied chemotherapy, the identification of new plant compounds represents a huge potential for research. It is important that most plant ingredients are safe to use [6,7].

In the last 15 years, there has been a growing interest in the polyphenolic components found in plants of the genus *Centaurea* L. (Asteraceae). It is a large genus, including several hundred representatives, originating from the Mediterranean basin (Turkey, Greece), but it is widespread on practically most of the continents, especially since the plants are quite expansive and do not require special soil conditions [8,9,10,11].

Many plants belonging to this genus have been tested for therapeutic properties. These pieces of research included essential oils as well as flavonoids that occurred in tested herbs. Studies have revealed that extracts from plants belonging to the genus *Centaurea* L. act as digestive enhancers, stimulants of the production and secretion of bile and lowering agents for blood pressure. Due to the high availability and common nature of these plants, they are often used in folk medicine as antidiarrheal, astringent, antipyretic, anti-inflammatory, and anti-rheumatic agents, as well as antifungal, antibacterial, and antidiabetic agents. They also have valuable compounds with strong antioxidant and even anti-cancer potential [7,12,13,14,15]. 

One of the least known and described plants of the genus *Centaurea* L. is *Centaurea castriferrei* Borbás & Waisb. It comes from the Vas region (Hungary) and is a highly endemic species. It is probably a cross of *Centaurea stenolepis* A. Kerner subsp. *stenolepis* (*C. cetia* (G. Beck) H. Wagner, from *Centaurea pseudophrygia* subsp. *pseudophrygia* (CA Meyer) Gugler [16].

However, this species has not yet been described either in terms of its chemical composition or its potential for biological activity. Therefore, the aim of the study was the phytochemical analysis of methanol–water (7:3 *v*/*v*) and water extracts obtained from the blooming aerial parts of the *C. castriferrei*, as well as evaluation of their anticancer activity.

## 2. Results and Discussion

### 2.1. Determination of Total Phenolic Content and Total Flavonoid Content

**Figure 1:**
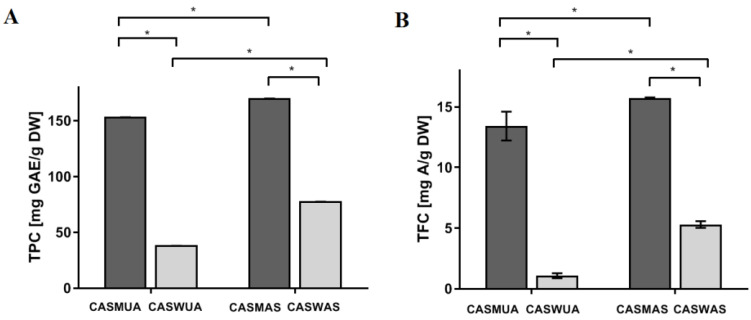
(**A**) Total Phenolic Content (TPC) and (**B**) Total Flavonoid Content (TFC) of different *C. castriferrei*. extracts. CASMUA: *Centaurea castriferrei* Borbás & Waisb ultrasound-assisted extraction methanol–water (7:3 *v*/*v*) extract; CASWUA: *Centaurea castriferrei* Borbás & Waisb ultrasound-assisted extraction water extract; CASMAS: *Centaurea castriferrei* Borbás & Waisb accelerated solvent extraction methanol–water (7:3 *v*/*v*) extract; CASWAS: *Centaurea castriferrei* Borbás & Waisb accelerated solvent extraction water extract. * *p* < 0.05.

As a result of the conducted extractions, a total of four extracts were obtained, and were determined as follows:

Methanol–water extracts of the herb (aerial part) of the investigated species contain significant amounts of polyphenols—CASMAS (*Centaurea castriferrei* Borbás & Waisb accelerated solvent extraction methanol–water (7:3 *v*/*v*) extract) (170.209 mg GAE/g plant substance), CASMUA (*Centaurea castriferrei* Borbás & Waisb ultrasound-assisted extraction methanol–water (7:3 *v*/*v*) extract) (153.604 mg GAE/g plant substance), while water extracts–CASWAS (*Centaurea castriferrei* Borbás & Waisb accelerated solvent extraction water extract) (77.822 mg GAE/g plant substance) and CASWUA (*Centaurea castriferrei* Borbás & Waisb ultrasound-assisted extraction water extract) (38.296 mg GAE/g plant substance) of these compounds are much less (Figure 1A; Table 1 and Table 2).

As in the case of the content of polyphenols, the highest content of flavonoids was found in methanol–water extracts obtained both with the Accelerated Solvent Extraction (ASE) and Ultrasound-Assisted Extraction (UAE) techniques—CASMAS (15.748 mg A/g of plant substance) and CASMUA (13.418 mg A/1 g of the plant substance) (Figure 1B; Table 3).

### 2.2. Determination of the Ability to Reduce Copper Ions (CUPRAC)

**Figure 2:**
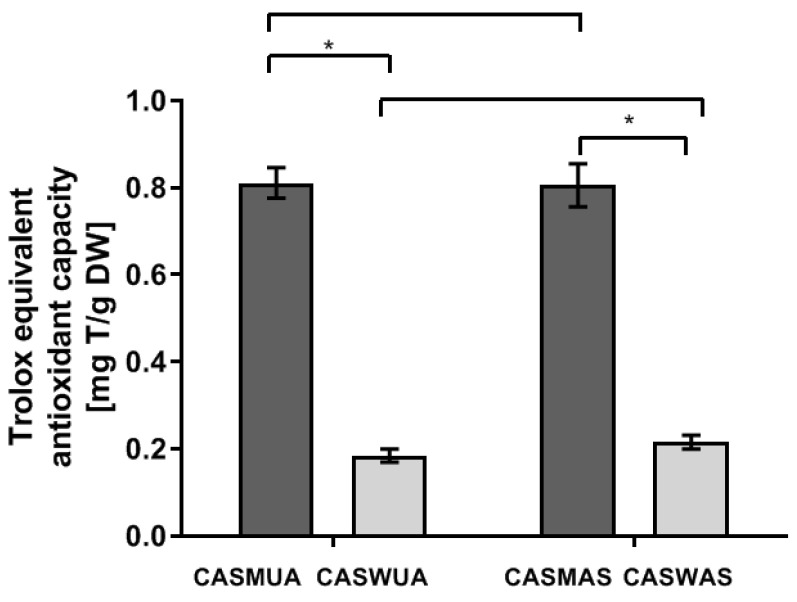
Average equivalent antioxidant capacity of Trolox in the tested extracts by the CUPRAC method (mg T/g of plant substance). CASMUA: *Centaurea castriferrei* Borbás & Waisb ultrasound-assisted extraction methanol–water (7:3 *v*/*v*) extract; CASWUA: *Centaurea castriferrei* Borbás & Waisb ultrasound-assisted extraction water extract; CASMAS: *Centaurea castriferrei* Borbás & Waisb accelerated solvent extraction methanol–water (7:3 *v*/*v*) extract; CASWAS: *Centaurea castriferrei* Borbás & Waisb accelerated solvent extraction water extract. * *p* < 0.05.

The methanol–water extracts had higher antioxidant activity than the water ones, regardless of the extraction method used (Figure 2). The ability to reduce copper ions was comparable in both methanol–water extracts—CASMUA and CASMAS—as well as in both CASWUA and CASWAS water extracts (Table 4). The results showed a correlation between the antioxidant activity (CUPRAC) and the total content of phenols and flavonoids. The high total phenolic and total flavonoid content can contribute to the high potential antioxidant activities of methanol–water extracts.

### 2.3. Qualitative and Quantitative Analysis

#### 2.3.1. RP-HPLC/DAD Analysis

RP/HPLC analysis for methanol–water and water extracts using two extraction techniques was presented for the first time. To date, no information on the composition of flavonoid compounds or phenolic acids has been found in the available literature. RP-HPLC/DAD analysis (Table 5) using available standards showed that the main constituents of the extracts are apigenin (the highest content in the studied extracts) and its derivatives: 7-O-glucuronide (the second most abundant component of the extract) and apigenin 7-O-glucoside or dimethyl apigenin, and others. In addition, luteolin 7-O-glucoside and kaempferol derivatives (especially dihydrokaempferol) are also present. Centaurein and jacein were also identified in the extracts studied. These are compounds identified in the Asteraceae family, found primarily in the genus *Centaurea* L., but are not present in all species. Centaurein in alcoholic extracts is the third most abundant compound in the group of flavonoids present in the extracts studied. Determining phenolic acids, chlorogenic acid, and its derivatives (chlorogenic acid glucoside, crypto-chlorogenic acid, neochlorogenic acid) proved to be the dominant component, especially in methanol–water extracts. Caffeic acid and its derivatives are also an important group. In addition, protocatechuic acid and 4-hydroxybenzoic acid are among the more important components in terms of quantity. 

The totals of identified components in all the extracts tested indicate that the methanol–water extracts contain more components in mg/g of plant material d.wt. (28.63 CASMUA and 29.48 CASMAS, respectively) than their water-based counterparts (6.07 CASWUA and 17.33 CASWAS), except for cynarin 1.3, identified only in the CASWUA extract and apigenin determined at the highest concentration in the CASWAS extract.

#### 2.3.2. Qualitative Analysis—LC/ESI-QTOF-MS 

Qualitative analysis revealed that the dominant/predominant components of the examined extracts are apigenin and its derivatives, e.g., glucuronide. Table 6, Table 7, Table 8 and Table 9 show the qualitative composition of the examined extracts.

### 2.4. Cytotoxicity of Extracts

**Figure 3:**
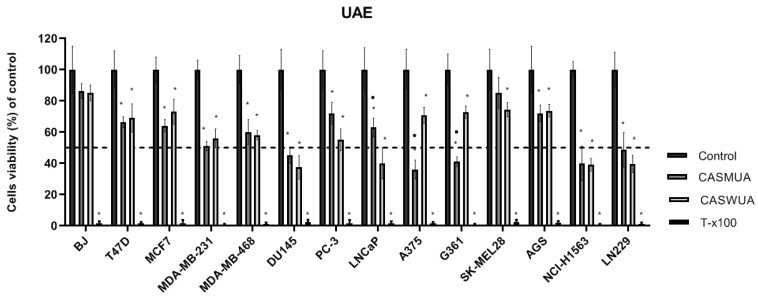
Relative viability of tested cell lines treated with two extracts of *Centaurea castriferrei* Borbás & Waisb. obtained with the UAE method in the highest concentration used (CASMUA/CASWUA—125 μg/mL both), DMSO as a vehicle in control cultures, and 1% T-x100-containing medium as a positive control for 48 h determined by MTT assay. The results were calculated as % of control cultures’ viability which were averaged to define the 100%. Values were presented as mean ± SD derived from three independent experiments. CASMUA/CASWUA vs. Control * *p* < 0.05; CASMUA vs. CASWUA • *p* < 0.05. UAE: ultrasound-assisted extraction; CASMUA: *Centaurea castriferrei* Borbás & Waisb ultrasound-assisted extraction methanol–water (7:3 *v*/*v*) extract; CASWUA: *Centaurea castriferrei* Borbás & Waisb ultrasound-assisted extraction water extract; T47D, MCF-7, MDA-MB231, MDA-MB468: human breast cancer cell lines; PC-3, DU145, LNCaP: human prostate cancer cell lines; A375, G361, SK-MEL28: human malignant melanoma cell lines; LN229: human glioblastoma cell line; AGS: human gastric adeno-carcinoma cell line; NCI-H1563: human non-small cell lung cancer cell line; BJ: human normal fibroblast cell line; T-x100: Triton-x100.

**Figure 4:**
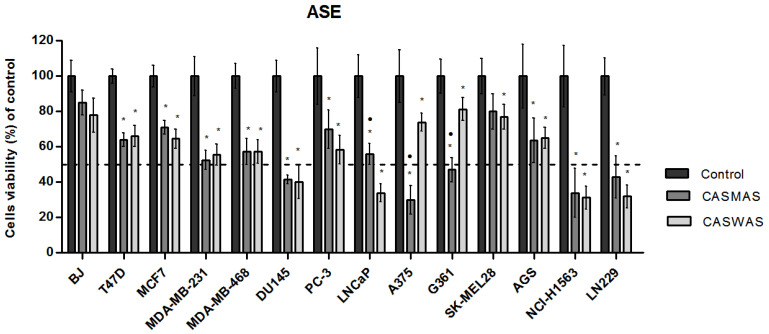
Relative viability of tested cell lines treated with two extracts of *Centaurea castriferrei* Borbás & Waisb. Obtained with the ASE method in the highest concentration used (CASMAS/CASWAS—125 μg/mL both) or DMSO as a vehicle in control cultures for 48 h determined by MTT assay. The results were calculated as % of control cultures viability which were averaged to define the 100%. Values were presented as mean ± SD derived from three independent experiments. CASMAS/CASWAS vs. Control * *p* < 0.05; CASMAS vs. CASWAS • *p* < 0.05. ASE: accelerated solvent extraction; CASMAS: *Centaurea castriferrei* Borbás & Waisb accelerated solvent extraction methanol–water (7:3 *v*/*v*) extract; CASWAS: *Centaurea castriferrei* Borbás & Waisb accelerated solvent extraction water extract; T47D, MCF-7, MDA-MB231, MDA-MB468: human breast cancer cell lines; PC-3, DU145, LNCaP: human prostate cancer cell lines; A375, G361, SK-MEL28: human malignant melanoma cell lines; LN229: human glioblastoma cell line; AGS: human gastric adenocarcinoma cell line; NCI-H1563: human non-small cell lung cancer cell line; BJ: human normal fibroblast cell line.

The antiproliferative activity of the obtained four extracts (Figure 3 and Figure 4) was tested in thirteen cancer cell lines: human breast cancer cell lines (MCF7, T47D, MDA-MB-231, MDA-MB-468); human prostate cancer cell lines (PC-3, DU145, LNCaP); human melanoma cell lines (A375, G361, SK-MEL28); human glioblastoma cell line (LN229); human gastric adenocarcinoma cell line (AGS); human non-small cell lung cancer cell line (NCI-H1563); and human normal fibroblast cell line (BJ). The cells were treated with extracts in a wide range of concentrations (max. conc. 125 μg/mL). The effect of the extracts on cancer cell viability was variable. In maximal concentration, the tested extracts possessed a cytotoxic effect against all tested cell lines and did not cause a significant decrease in the viability of BJ cells.

Regardless of the extraction method and the extractant used, similar cytotoxicity of the extracts on most cancer cell lines was observed.

Only in the case of A375 and G361 melanoma cell lines, were the methanol–water (CASMUA/CASMAS) extracts more effective (cytotoxic) as compared to water extracts (CASWUA/CASMAS). In turn, in the case of the LNCaP cell line, it was noted that water extracts possessed stronger cytotoxic properties as compared to the methanol–water extracts. IC_50_ values for extracts were determined using the AAT Bioquest IC50 calculator. In Table 10, the IC_50_ values for extracts in testing cell lines are shown.

A hallmark of cells in many types of cancers is an imbalance between ROS production and antioxidant defense. Increased levels of ROS, which result from metabolic disturbances at the cellular level and impaired signal transduction, promote carcinogenesis and tumor progression through the activation of oncogenes [17,18,19]. On the other hand, further increases in ROS levels in cancer cells can induce irreversible changes and consequent apoptosis—this is how a number of anticancer drugs work, e.g., cisplatin or anthracyclines [20,21]. Taking into account the greater antioxidant properties of the methanol–water extracts and the lack of differences in cytotoxic activity in cancer cell lines compared to water extracts, it turned out that their antitumor activity did not correlate with their strong/weak antioxidant properties. However, since oxidative stress plays an important role in carcinogenesis and cancer progression, the compounds of the methanol–water extracts have the potential to exhibit chemopreventive activity, which could be investigated in the future.

The water extracts had fewer phenolic components, but the cytotoxic activity against the tested cell lines varied and seemed to depend more on the type of cell line. e.g., in the case of prostate cancers, the water extracts had stronger activity than the methanolic ones. Given that all extracts showed cytotoxic activity against most of the cell lines tested, and it did not depend on the amount of compounds contained, the composition of the extracts was analyzed for compounds that were present in all four types of extracts. We can state that this plant is a rich source of apigenin and its derivatives (the most amount of apigenin is contained in CASWUA; CASMUA and CASMAS contain more apigenin 7-O-glucuronide). Apigenin is a flavonoid commonly found in the diet and has strong anti-inflammatory, antioxidant, antimicrobial, and antiviral effects. Recent research has focused on the health-promoting effects of apigenin, so it can be used in dietary supplements [22]. Apigenin has been identified in the past in various species of the genus Centaurea: *Centaurea nicaeensis; Centaures urvillei; Centaurea cyanus; Centaurea jacea;* or *Centaurea scabiosa* [23,24,25,26]. The cytotoxic properties of apigenin against many cancers have been repeatedly described in the literature, including the tumors studied in this research. It has been revealed that apigenin acts by inhibiting the cell cycle, inducing apoptosis and autophagy of cancer cells [27,28,29]. Apigenin may also reduce the potential for cancer cells to migrate and invade [30].

Among the ingredients found in all four types of extracts, there are: chlorogenic acid and its derivatives, as well as centaurein, although their content is lower, especially in water extracts. Chlorogenic acid is primarily known for its strong antioxidant properties. It exhibits several biological activities—anti-inflammatory, anti-aging, anti-diabetic, and also anti-cancer. Chlorogenic acid has been shown to exert cytotoxic activity against lung, breast, colorectal, kidney, or glioma cancer cells [31,32,33,34,35]. Regarding centaurein, there are very few reports of its biological activity, and none relating to anticancer properties [36,37,38].

In qualitative analysis, hispidulin was identified in three types of extracts (except CAWUA). Many studies have shown the broad biological activity of this flavonoid, including anti-inflammatory and anti-cancer properties [39,40,41,42]. Hispidulin affects cell proliferation, apoptosis, cell cycle, and angiogenesis. Moreover, hispidulin exhibits synergistic anticancer effects in combination with common anticancer drugs, resulting in reduced chemotherapeutic efflux, increased chemosensitivity of cancer cells, and reversed drug resistance [43]. 

However, it seems unlikely that one particular component is responsible for the observed activity of the studied extracts. In the case of plant extracts, we can often observe a synergistic activity of the components [44]. For this reason, further research should identify the group of substances responsible for the observed biological effect.

## 3. Materials and Methods

### 3.1. Plant Material

The material for the research was obtained from the Botanical Gardens (Medicinal Plant Laboratory) of the Department of Pharmacognosy of the Medical University of Lublin. Harvesting took place from June to August (during the flowering of plants) in 2020. For experimental purposes, the above-ground parts (flowering herbs) of one species of the genus *Centaurea* L. were used: *C castriferrei*. (CAS). The seeds of this species were obtained as part of the seed exchange between the Botanical Gardens, and the first sowings were carried out in 2015–2017. The flowering aerial parts (40 cm from the top) of *C. castriferrei* were collected from the Botanical Garden (Medicinal Plant Laboratory) of the Department of Pharmacognosy of the Medical University of Lublin (22°33′50.868″ E, 51°15′22.8312″ N, 187 m above sea level) during August, 2020. The plant material was identified by Aleksandra Józefczyk PhD. Specimen *C. castriferrei*-2020 is deposited in the Department of Pharmacognosy. The collected flowering herb was dried in a forced-air dryer, not exceeding 30 °C, and then the plant material was stripped of the woody parts of the stems, leaving only the upper parts of the stems, leaves, and flowers. The herb prepared in this way was cut up and then ground in an electric grinder and sieved according to the requirements of Polish Pharmacopoeia XII (2020). The powdered plant matter was destined for further phytochemical and biological studies. 

### 3.2. Extraction of Plant Material

#### 3.2.1. Ultrasound-Assisted Extraction (UAE)

Extraction of plant material was carried out using an ultrasonic bath (Sonorex Bandelin, Germany). Exactly 20 g of powdered plant material was weighed, then transferred to 250 mL round-bottom flasks, and a portion of the extractant (120 mL) was added, stirring thoroughly. Two types of extractant were used in the study: water (distilled) and a mixture of methanol and water (7:3 *v*/*v*). Extractions were carried out for 30 min at 65 °C. After this time, the cooled extract was filtered into a round-bottom flask (1000 mL), and the remaining plant material was extracted again under identical conditions. The operations were repeated once more. The resulting methanol–water extract was distilled to dryness and re-dissolved in this extract by transferring to a 100 mL volumetric flask. The water extract was concentrated and transferred to a 100 mL volumetric flask.

#### 3.2.2. Accelerated Solvent Extraction (ASE)

For the study, samples of plant material (1.0 g) were prepared and placed in the extraction cell of an ASE 100 device (Accelerated Solvent Extraction; Dionex, Sunnyvale, CA, USA). Solvents (methanol–water mixture 7:3 *v*/*v* and distilled water) were used as extractant. Extraction of plant material was carried out with the following process conditions: pressure—100 bar; temperature—65 °C; condition time—5 min; wash volume—60%; purification time—60 s; number of cycles—3. The operations were repeated 5 times. After extraction, solvents were distilled from the obtained extracts, and the dry residue was dissolved again and transferred to 25 mL volumetric flasks, obtaining for the tested extracts the same initial concentrations as for the extracts using ultrasound, i.e., 2 g/10 mL of extract.

#### 3.2.3. Solid Phase Extraction (SPE)

Solid Phase Extraction (SPE) was used to purify the extracts from chlorophyll and impurities. The method used a reduced-pressure filter chamber (J.T. Baker, Phillipsburg, NJ, USA) and micro-columns (500 mg) for SPE filled with octadecyl (C18) bed as a stationary phase. Due to the differences in the extractants used, there were two approaches in the extraction method relating to the eluent used: for methanol–water (7:3 *v*/*v*) extracts, the same solvent was used in the purification process; for water extracts, a methanol–water (1:9 *v*/*v*) mixture was used to elute the active ingredients. 

The extraction process itself to the solid phase was divided into two parts: conditioning of the stationary phase of the column (sorbent) and the main process of sample purification. In sample preparation, 10 mL of methanol was used and then filtered under vacuum −0.01 MPa until the sorbent was dry, and then, depending on the type of extract, 10 mL of the methanol–water mixture (7:3 *v*/*v*) or methanol–water mixture (1:9 *v*/*v*) was applied to the bed and filtered under pressure −0.01 MPa until a residue of 1 mm of liquid above the bed. Then, 2 mL of the test extract was applied to the wetted sorbent and then washed with portions of the appropriate solvent into 10 mL flasks. Extracts were obtained with compounds isolated from 0.4 g of plant material/10 mL of extract.

### 3.3. Chemical Analysis

#### 3.3.1. Determination of Flavonoid Compounds (TFC)

For determining the content of flavonoid compounds (Total Flavonoids Content) in the extracts studied, a spectrophotometric method was used, taking advantage of the ability to form color complexes of these compounds with +3 metal ions (e.g., aluminum. iron). Before the determinations, the stock solutions (extracts) were diluted 9 times. A total of 100 μL of extract (triplicates) and 100 μL of 2% aluminum chloride solution (AlCl_3_) were applied to 96-well plates. Plates were incubated for one hour in the absence of light, and then absorbance was measured at λ = 415 nm using a plate reader (Epoch) with a control station and Gen5 Data Analysis Software version 3.08 (BioTek, Winooski, VT, USA). Test extracts (100 µL) mixed with methanol (100 µL) were used as references. The blanks in the assay were wells filled with 200 µL of methanol. 

Based on the standard curve for reference substance—apigenin (y = 0.0102x + 0.0045)—the total flavonoid content was calculated using a modification of the formula proposed by Alara et al., 2020 [45]:

TFC [mg A/1 g plant material] = c × V/m

where c—sample concentration read from the calibration curve (mg/mL); V—solvent volume (mL); sample mass (mg).

#### 3.3.2. Determination of Total Polyphenols (TPC) 

A spectrophotometric method was also used to determine the total phenolic content. The stock extract was diluted 10-fold, and then 10 µL of each extract was applied to 96-well plates (Epoch) in triplicate. Then, 100 µL of Folin-Ciocalteu reagent (FCR) was added. After 5 min, 100 µL of 7.5% Na_2_CO_3_ was added to each sample. The samples were incubated for 1 h without light. The following were used as reference: test extract (10 μL); 7.5% Na_2_CO_3_ solution (100 μL); and 100 μL methanol was added instead of the color-inducing reagent. The blank was methanol (210 μL). The reaction resulted in a color change of the reagent from yellow to blue. Gallic acid solution (GAE) was used as a conversion standard—the concentration of the stock solution was 100 mg/100 mL of water. The absorbance of the test solutions (λ = 760 nm) was then measured using Gen5 Data Analysis Software version 3.08 (BioTek Instruments, Winooski, VT, USA). A standard curve (y = 0.0515x + 0.0185) was plotted for gallic acid, from which the values for each extract were determined. Total phenolic content expressed in mg GAE equivalents/g of extract was calculated according to the formula proposed by Alara et al., 2020 [45]:

TPC [mg GAE/g of plant material] = c × V/m

where c—sample concentration read from the calibration curve (mg/mL); V—solvent volume (mL); m—sample weight (mg).

#### 3.3.3. RP-HPLC/DAD Analysis

Tested extracts analysis were performed using an Agilent Technologies (Waldbronn, Germany) 1100 Series liquid chromatograph with a visible diode–array detector (DAD) and an autosampler using the ChemStation software (Agilent Technologies, Santa Clara, CA, USA). Chromatographic separation of examined extracts was performed on a Zorbax Eclipse XDB C8 column (150 × 4.6 mm, dp = 5 μm) working at 25 °C with gradient elution: A–water with 1% (*v*/*v*) acetic acid; B–acetonitrile (0 min. 10% B; 0–10 min. 10–15% B; 10–15 min. 15–22% B; 15–25 min. 22–30% B; 25–35 min. 30–35% B; 35–45 min. 35–55% B; 45–57 min. 55–90% B). The flow rate of a mobile phase was 1 mL/min and the injection volume was 10 μL for extracts and standards. To equilibrate the chromatographic column after ending each analysis, a post-run time of 20 min was additionally introduced to the gradient program. The identification of the compounds in extracts was performed by comparing retention times and UV spectra (λ = 254, 280, and 325 nm) with those parameters obtained for the reference compounds analyzed under identical chromatographic separation conditions. The methodology used was created for the broader panel of studies among whom the studies described in this paper were conducted. A quantitative analysis of the polyphenolic compounds was performed based on the external standard method. Analysis of each extract was performed in 3-fold determinations, which allowed for a statistical evaluation of the obtained results. 

#### 3.3.4. HPLC/ESI-QTOF-MS

Extended qualitative determination of the polyphenolic components of the tested extracts was performed by an HPLC/ESI-QTOF-MS system in positive ion mode using a 6530B Accurate-mass-QTOF-LC/MS (Agilent Technologies Inc., Santa Clara, CA, USA) mass spectrometer with an ESI-Jet Stream ion source. The Agilent 1260 chromatograph was equipped with autosampler, DAD detector, binary gradient pump, and column oven. The separation was achieved by a Gemini C18 column (100 × 2.1 mm dp = 3 µm) (Phenomenex Inc., Torrance, CA, USA) and heated to 25 °C. The mobile phases consisted of water with 0.1% acetic acid and 1% acetonitrile (solvent A) and acetonitrile with the addition of 0.1% acetic acid and 5% deionized water (solvent B). The following gradient was applied: 0 min 15% B; 35 min 25% B; 65 min 95% B. The post time was 12 min. Injection volume for extracts was 10 μL. The flow rate was set at 0.2 mL/min and total time of analysis was 65 min. The UV spectra were recorded at wavelengths λ = 254, 270, and 320 nm. Analysis was performed with the other following parameters: gas temperature 300 °C, gas flow 12 L/min, nebulizer pressure: 35 psig; sheath gas temperature 325 °C; sheath gas flow 12 mL/min; capillary voltage 4000 V, skimmer 65 V; fragmentor 140 V; octopole RF Peak: 750 V, mass range 100–1000 m/z, scan range 1 spectrum per s. Identification of compounds was based on comparison of the data with the literature, our own collected data, and the METLIN database.

#### 3.3.5. Copper Reduction Assay (CUPRAC)

The cupric ion-reducing antioxidant capacity (CUPRAC) assay is based on measuring the use of copper (II)-neocuproine (2,9-dimethyl-1,10-phenanthroline) [Cu (II)-Nc] as a chromogenic oxidizing agent. The determinations were performed by modifying the method proposed by Apak et al. [46] for 96-well plates. Nine dilutions of extracts were used for analysis. Samples were prepared by mixing 40 μL of test extract, 50 μL of 10^−2^ M copper (II) chloride dihydrate, 50 μL of 7.5 × 10^−3^ methanolic neocuproine solution, and 60 μL of ammonium acetate buffer (pH 7.0) in each well. After 30 min of incubation at room temperature (25 °C), the absorbance of the prepared mixtures was measured at =450 nm using an Epoch microplate spectrophotometer with a control station and Gen5 Data Analysis Software, Version 3.08 (BioTek Instruments, Winooski, VT, USA). For each dilution of the extract, 3 replicates (*n*-3) were performed. Based on the results, a standard curve was drawn for the standard Trolox (y = 1.9695x + 0.05). The equivalent antioxidant capacity of Trolox was calculated. using the formula:

TEAC [mg trolox/1 g plant substance] = c × V/m

where c—sample concentration read from the calibration curve (mg/mL); V—solvent volume (mL); sample mass (mg).

### 3.4. Biological Activity 

#### 3.4.1. Cell Culturing

The research was conducted on the following cell lines: human breast cancer cell lines (MCF7, T47D, MDA-MB-231, MDA-MB-468); human prostate cancer cell lines (PC-3, DU145, LNCaP); human melanoma cell lines (A375, G361, SK-MEL28); human glioblastoma cell line (LN229); human gastric adenocarcinoma cell line (AGS); human non-small cell lung cancer cell line (NCI-H1563); and human normal fibroblast cell line (BJ). All the cell lines were obtained from American Type Culture Collection (ATCC, Manassas, VA, USA). The cells were maintained in media (Corning, New York, NY, USA) selected in accordance with ATCC recommendations supplemented with 10% fetal bovine serum (Life Technologies, Carlsbad, CA, USA) and antibiotics such as penicillin (100 units) and streptomycin (100 µg/mL) (Sigma-Aldrich, St. Louis, MO, USA). The cells were cultured in a humidified atmosphere of 5% CO_2_ at 37 °C.

#### 3.4.2. The Cytotoxicity Analysis

The cytotoxicity of tested extracts was measured by performing MTT assay using the MTT Cell Proliferation Assay Kit (Invitrogen, Waltham, MA, USA). The test is based on the ability of living cells to reduce orange tetrazolium salt by cellular dehydrogenases. to water-insoluble purple formazan crystals. Cells were seeded into 96-well plates in the concentration range from 1 × 10^5^ cells/mL to 2.5 × 10^5^ cells/mL depending on the used cell line. Stock solutions for cytotoxicity analysis were prepared by dissolving 50 mg of dry plant extracts in 1 mL of DMSO. For biological activity studies, extracts not purified by SPE were used. Tested extracts were added to the cell cultures when 70–80% of confluence was achieved in a wide range of concentrations (maximum concentration 125 µg/mL). MTT solution (4 mg/mL) was added to the culture after 48 h of incubation with tested extracts. Following 4 h of incubation, the medium with MTT salt was removed and obtained crystals were dissolved in DMSO (dimethyl sulfoxide, 200 mL/well; POCH, Poland). The solution absorbency was measured at 570 nm using PowerWave™ microplate spectrophotometer (Bio-Tek Instruments, Winooski, VT, USA). Each cytotoxicity assay was conducted three times and was measured in triplicates.

## 4. Conclusions

Researching plant extracts to discover their therapeutic activity is constantly of interest to scientists. In this study, the phytochemical analysis of the plant *C. castriferrei* was performed for the first time. The conducted research has shown that methanol–water extracts (7:3 *v*/*v*) contain greater amounts of polyphenols and flavonoids compared to water extracts. Qualitative and quantitative analysis indicated that *C. castriferrei* is a rich source of apigenin and its derivatives. Chlorogenic acid and centaurein were also identified in all tested extracts. In vitro studies have indicated the cytotoxic activity of both methanol–water (7:3 *v*/*v*) and water extracts against many cancer cell lines, e.g., prostate cancer, lung cancer, and glioblastoma. This indicates that the plant may be a potential source of compounds with anticancer activity. Furthermore, it is relevant to consider compounds of plant extracts as adjuvant agents in cancer therapy. Therefore, in future studies, we plan to isolate the compound or a group of compounds acting synergistically that are responsible for the observed biological effect. Comparative analysis showed that both water and methanol–water extracts (7:3 *v*/*v*) showed similar biological effects. However, given the amount of isolated phenolic compounds, in future studies, it is worth using methanol as an extractant. Similarly, both isolation methods, UAE and ASE, showed similar efficiencies; however, due to the greater availability of UAE, it appears to be more beneficial.

## Figures and Tables

**Table 1:** The Extracts’ Abbreviations Used in the Research.

Species	Ultrasound Assisted Extraction (UAE)		Accelerated Solvent Extraction (ASE)	
	Methanol-Water (7:3 *v*/*v*)	Water	Methanol-Water (7:3 *v*/*v*)	Water
***C. castriferrei*** **Borbás & Waisb.**	CASMUA	CASWUA	CASMAS	CASWAS

**Table 2:** Determination of Polyphenols (TPC) Content in Extracts from *C. castriferrei*.

Extract	Polyphenol Content from the Standard Curve (μg/mL)	Polyphenol Content/ GAE Equivalent per g Plant Substance (mg GAE/g)	Mean Content (mg GAE/g)	±SD; ±RSD
**CASMUA**	14.63 7.31 3.66	153.56 153.53 153.73	153.60	SD ± 0.10 RSD ± 0.08
**CASWUA**	3.65 1.82 0.92	38.27 38.21 38.41	38.30	SD ± 0.11 RSD ± 0.08
**CASMAS**	16.19 8.12 4.05	170.04 170.55 170.04	170.21	SD ± 0.29 RSD ± 0.23
**CASWAS**	7.40 3.70 1.86	77.74 77.68 78.05	77.82	SD ± 0.20 RSD ± 0.15

CASMUA: *Centaurea castriferrei* Borbás & Waisb ultrasound-assisted extraction methanol–water (7:3 *v*/*v*) extract; CASWUA: *Centaurea castriferrei* Borbás & Waisb ultrasound-assisted extraction water extract; CASMAS: *Centaurea castriferrei* Borbás & Waisb accelerated solvent extraction methanol–water (7:3 *v*/*v*) extract; CASWAS: *Centaurea castriferrei* Borbás & Waisb accelerated solvent extraction water extract.

**Table 3:** Determination of Flavonoids (TFC) Content in Extracts from *C. castriferrei*.

Extract	Flavonoid Content from the Standard Curve (μg/mL)	Flavonoid Content/ A (Apigenin) Equivalent per g Plant Substance (mg A/g)	Mean Content (mg A/g)	±SD; ±RSD
**CASMUA**	72.06 34.36 15.12	14.41 13.75 12.10	13.42	SD ± 1.19 RSD ± 0.88
**CASWUA**	6.49 2.21 1.32	1.30 0.88 1.06	1.09	SD ± 0.21 RSD ± 0.15
**CASMAS**	78.63 39.34 19.73	15.73 15.74 15.78	15.75	SD ± 0.03 RSD ± 0.02
**CASWAS**	25.59 14.02 6.45	5.12 5.61 5.16	5.30	SD ± 0.27 RSD ± 0.21

CASMUA: *Centaurea castriferrei* Borbás & Waisb ultrasound-assisted extraction methanol–water (7:3 *v*/*v*) extract; CASWUA: *Centaurea castriferrei* Borbás & Waisb ultrasound-assisted extraction water extract; CASMAS: *Centaurea castriferrei* Borbás & Waisb accelerated solvent extraction methanol–water (7:3 *v*/*v*) extract; CASWAS: *Centaurea castriferrei* Borbás & Waisb accelerated solvent extraction water extract.

**Table 4:** Determination of Antioxidant Properties with the Use of Copper Ions in the Tested Extracts of *C. castriferrei*.

Extract	Trolox Concentration from the Standard Curve (mg/mL)	Trolox Equivalent Antioxidant Capacity (mg T/g .d.wt.)	Mean Content (mg T/g .d.wt.)	±SD; ±RSD
**CASMUA**	0.39 0.21 0.10 0.05 0.03	0.78 0.83 0.81 0.78 0.86	0.81	SD ± 0.03 RSD ± 0.03
**CASWUA**	0.35 0.19 0.10 0.05 0.02	0.17 0.19 0.21 0.19 0.17	0.18	SD ± 0.01 RSD ± 0.01
**CASMAS**	0.38 0.20 0.11 0.05 0.10	0.75 0.79 0.86 0.86 0.77	0.81	SD ± 0.05 RSD ± 0.04
**CASWAS**	0.42 0.23 0.12 0.05 0.03	0.21 0.23 0.24 0.21 0.20	0.22	SD ± 0.02 RSD ± 0.01

CASMUA: *Centaurea castriferrei* Borbás & Waisb ultrasound-assisted extraction methanol–water (7:3 *v*/*v*) extract; CASWUA: *Centaurea castriferrei* Borbás & Waisb ultrasound-assisted extraction water extract; CASMAS: *Centaurea castriferrei* Borbás & Waisb accelerated solvent extraction methanol–water (7:3 *v*/*v*) extract; CASWAS: *Centaurea castriferrei* Borbás & Waisb accelerated sol-vent extraction water extract.

**Table 5:** Identification and Quantification (mg/g dry weight (d.wt.)) of the Content of the Ingredients of the Tested CASMUA, CASWUA, CASMAS, CASWAS Extracts Obtained from the Aerial Parts of *C. castriferrei* Borbás & Waisb.

No.	Name of Compound	CASMUA			CASWUA			CASMAS			CASWAS		
		Content (mg/g)			Content (mg/g)			Content (mg/g)			Content (mg/g)		
			±SD	±RSD		±SD	±RSD		±SD	±RSD		±SD	±RSD
**1**	Neochlorogenic acid	**0.90**	0.01	0.6	**0.51**	0.00	0.2	**1.02**	0.00	0.4	**0.14**	0.00	2.5
**2**	Chlorogenic acid	**4.14**	0.03	0.7	**1.30**	0.01	0.5	**4.06**	0.01	0.3	**0.17**	0.00	0.2
**3**	Crypto-chlorogenic acid	**0.11**	0.00	1.7	**0.04**	0.00	0.0	**0.11**	0.00	0.0	-	-	-
**4**	Caffeic acid	**0.09**	0.00	0.7	**0.09**	0.00	1.0	**0.06**	0.00	1.0	-	-	-
**5**	Protocatechuic acid	**0.26**	0.01	3.5	**0.17**	0.00	2.2	**0.31**	0.01	3.2	**0.07**	0.00	0.0
**6**	4-Hydroxybenzoic acid	**0.15**	0.00	0.8	-	-	-	**0.17**	0.00	0.4	**0.20**	0.00	1.4
**7**	Isoferulic acid	**0.02**	0.00	0.8	-	-	-	**0.02**	0.00	1.7	-	-	-
**8**	*p*-Coumaric acid	-	-	-	-	-	-	-	-	-	**0.04**	0.00	0.0
**9**	Cynarin 1.3	-	-	-	**0.38**	0.00	0.6	-	-	-	-	-	-
**10**	Chlorogenic acid glucoside	**1.70**	0.00	0.1	**0.14**	0.00	0.9	**1.91**	0.02	1.2	**0.04**	0.00	0.0
**11**	Caffeic acid derivative 1	**0.16**	0.00	1.4	**0.08**	0.00	0.4	**0.17**	0.00	1.3	**0.07**	0.00	0.9
**12**	Caffeic acid derivative 2	**0.08**	0.00	0.6	-	-	-	**0.08**	0.00	0.0	**0.04**	0.00	0.7
**13**	Caffeic acid derivative 3	**0.11**	0.00	0.5	-	-	-	**0.14**	0.00	0.4	-	-	-
**14**	Apigenin derivative	**0.40**	0.01	1.3	**0.21**	0.00	0.2	**0.41**	0.01	1.5	**0.37**	0.00	0.0
**15**	Isoquercetin	**0.05**	0.00	0.9	-	-	-	**0.05**	0.00	1.1	-	-	-
**16**	Luteolin 7-*O*-glucoside	**0.85**	0.01	0.6	**0.51**	0.01	2.4	**0.62**	0.02	2.6	**0.39**	0.00	0.3
**17**	Apigenin 7-*O*-glucoside	**0.17**	0.00	0.4	-	-	-	**0.17**	0.00	0.4	-	-	-
**18**	Apigenin 7-*O*-glucuronide	**5.26**	0.02	0.3	**1.76**	0.01	0.3	**5.26**	0.02	0.3	**2.62**	0.01	0.2
**19**	Dimethyl apigenin	**0.56**	0.00	0.3	**0.11**	0.00	0.6	**0.60**	0.00	0.7	**0.14**	0.00	0.4
**20**	Dihydrokaempferol	**0.82**	0.02	0.0	**0.23**	0.00	0.0	**0.92**	0.03	2.8	**0.36**	0.00	0.0
**21**	Kaempferol dihydro- glucoside	**0.09**	0.00	0.0	-	-	-	**0.09**	0.00	0.0	-	-	-
**22**	Kaempferol glucoside	**0.08**	0.00	0.0	-	-	-	**0.11**	0.00	0.0	-	-	-
**23**	Centaurein	**3.97**	0.02	0.4	**0.16**	0.00	0.6	**4.11**	0.01	0.2	**0.76**	0.00	0.4
**24**	Jacein	**1.21**	0.00	0.2	-	0	0	**0.97**	0.00	0.3	**0.43**	0.00	1.1
**25**	Apigenin	**7.32**	0.03	0.4	**0.38**	0.01	2.6	**7.98**	0.02	0.3	**11.09**	0.01	0.1
**26**	Luteolin	**0.13**	0.00	0.0	-	-	-	**0.14**	0.00	0.0	**0.03**	0.00	0.0
**27**	Centaureidin	-	-	-	-	-	-	-	-	-	**0.51**	0.1	0.0
**Total (identified compounds)**		**28.63**			**6.07**			**29.48**			**17.33**		

CASMUA: *Centaurea castriferrei* Borbás & Waisb ultrasound-assisted extraction methanol–water (7:3 *v*/*v*) extract; CASWUA: *Centaurea castriferrei* Borbás & Waisb ultrasound-assisted extraction water extract; CASMAS: *Centaurea castriferrei* Borbás & Waisb accelerated solvent extraction methanol–water (7:3 *v*/*v*) extract; CASWAS: *Centaurea castriferrei* Borbás & Waisb accelerated sol-vent extraction water extract.

**Table 6:** CASMUA Extract. Fragmentation Analysis of Identified Compounds.

No.	Name of Compound	R_t_ (min)	Chemical Formula	Molecular Ion (m/z)	MS/MS Fragments (m/z)
**1**	Chlorogenic acid	15.735	C_16_H_18_O_9_	353.0846	191.0542
**2**	Feruloylquinic acid	20.158	C_17_H_20_O_9_	367.0989	191.0531; 134.0258; 93.0413
**3**	Apigenin glucuronide-glucoside	21.429	C_27_H_28_O_16_	607.1286	431.0946; 269.0409; 175.0196; 113.0224
**4**	Kaempferide glucoside	23.376	C_22_H_22_O_11_	463.0861	301.0397; 151.0012; 97.3310
**5**	Isorhamnetin glucoside	24.507	C_22_H_22_O_12_	477.1000	315.0631
**6**	Chlorogenic acid glucoside	25.911	C_21_H_28_O_24_	515.1150	353.0852; 191.0536
**7**	Isorhamnetin glucuronide	26.546	C_22_H_20_O_13_	491.1154	315.0631
**8**	Apigenin 7-*O* glucuronide	27.153	C_21_H_18_O_11_	445.0736	269.0441; 175.0241; 113.0202
**9**	Centaurein	27.455	C_24_H_26_O_13_	521.1231	506.1033; 343.0375
**10**	Jacein	27.960	C_24_H_26_O_13_	521.1240	506.1076; 359.0687; 343.0444
**11**	Hispidulin glucuronide	28.282	C_22_H_20_O_12_	475.0842	299.0494; 284.0258; 255.0054; 227.0327; 85.0249
**12**	Isorhamnetin	30.863	C_16_H_12_O_7_	315.0470	300.0327; 199.0447; 65.0458
**13**	Luteolin	31.090	C_15_H_10_O_6_	285.0475	133.0242; 107.0099
**14**	Apigenin	34.074	C_15_H_10_O_5_	269.0310	117.0348
**15**	Hispidulin	35.317	C_16_H_12_O_6_	299.0524	283.0272; 255.0443; 227.0484;

CASMUA: *Centaurea castriferrei* Borbás & Waisb ultrasound-assisted extraction methanol–water (7:3 *v*/*v*) extract.

**Table 7:** CASWUA Extract. Fragmentation Analysis of Identified Compounds.

No.	Name of Compound	R_t_ (min)	Chemical Formula	Molecular Ion (m/z)	MS/MS Fragments (m/z)
**1**	Quinic acid	1.865	C_7_H_12_O_6_	191.0525	111.0543
**2**	Protocatechuic acid glucoside	7.799	C_13_H_16_O_7_	153.0165	153.0154; 109.0277
**3**	Chlorogenic acid	10.087	C_16_H_18_O_9_	353.0863	191.0532; 179.0320
**4**	Neochlorogenic acid	15.646	C_16_H_18_O_9_	353.4838	191.0520; 135.0389; 85.0277
**5**	Feruloylquinic acid	20.186	C_17_H_20_O_9_	367.0987	191.0571; 134.0351; 93.0317
**6**	Ferulic acid	25.478	C_10_H_10_O_4_	193.0472	133.0283
**7**	Apigenin 7-*O* glucuronide	27.153	C_21_H_18_O_11_	445.0736	269.0440; 175.0241; 113.0202
**8**	Apigenin	34.138	C_15_H_10_O_5_	269.0439	117.0334

CASWUA: *Centaurea castriferrei* Borbás & Waisb ultrasound-assisted extraction water extract.

**Table 8:** CASMAS Extract. Fragmentation Analysis of Identified Compounds.

No.	Name of Compound	R_t_ (min)	Chemical Formula	Molecular Ion (m/z)	MS/MS Fragments (m/z)
**1**	Chlorogenic acid	15.829	C_16_H_18_O_9_	353.0827	191.0530
**2**	Feruloylquinic acid	20.198	C_17_H_20_O_9_	367.0987	191.0571; 134.0351; 93.0317
**3**	Isorhamnetin glucoside	24.512	C_21_H_18_O_13_	477.0994	315.0458; 299.0158
**4**	Chlorogenic acid glucoside	25.899	C_21_H_28_O_24_	515.1150	353.0836; 191.0506
**5**	Isorhamnetin glucuronide	26.569	C_22_H_20_O_13_	491.1184	315.7631
**6**	Apigenin 7-*O* glucuronide	27.199	C_21_H_18_O_11_	445.0756	269.0440; 175.0241; 113.0222
**7**	Hispidulin glucuronide	28.306	C_22_H_20_O_12_	475.0867	299.0544; 284.0308; 255.0254; 227.0327; 85.0289
**8**	Eriodictyol	29.846	C_15_H_12_O_6_	287.0517	151.0010; 135.0440
**9**	Isorhamnetin	30.893	C_16_H_12_O_7_	315.0490	300.0227; 199.0465; 65.0051
**10**	Luteolin	31.104	C_15_H_10_O_6_	285.0365	133.0269; 107.0092
**11**	Apigenin	34.109	C_15_H_10_O_5_	269.0439	117.0334
**12**	Hispidulin	35.304	C_16_H_12_O_6_	299.0545	283.0282; 255.0513; 227.0544;

CASMAS: *Centaurea castriferrei* Borbás & Waisb accelerated solvent extraction methanol–water (7:3 *v*/*v*) extract.

**Table 9:** CASWAS extract. Fragmentation Analysis of Identified Compounds.

No.	Name of Compound	R_t_ (min)	Chemical Formula	Molecular Ion (m/z)	MS/MS Fragments (m/z)
**1**	Coumaroylquinic acid	19.089	C_16_H_18_O_8_	337.0911	191.0517; 93.0339
**2**	Feruloylquinic acid	20.173	C_17_H_20_O_9_	367.1024	191.0533; 134.0361; 93.0341
**3**	Isorhamnetin glucuronide	26.544	C_22_H_20_O_13_	491.1184	315.7631
**4**	Apigenin *7-O* glucuronide	27.116	C_21_H_18_O_11_	445.0756	269.0440; 175.0241; 113.0222
**5**	Hispidulin glucuronide	28.272	C_22_H_20_O_12_	475.0867	299.0544; 284.0308; 255.0254; 227.0327; 85.0289
**6**	Isorhamnetin	31.142	C_16_H_12_O_7_	315.0490	300.0227; 199.0465; 65.0051
**7**	Apigenin	34.055	C_15_H_10_O_5_	269.0439	269.0442; 117.0334
**8**	Hispidulin	35.264	C_16_H_12_O_6_	299.0545	283.0542; 255.0513; 227.0544;

CASWAS: *Centaurea castriferrei* Borbás & Waisb accelerated solvent extraction water extract.

**Table 10:** The IC_50_ Values of Extracts in Testing Cell Lines.

Cell Lines		Extraction Method: ASE		Extraction Method: UAE	
		CASMAS	CASWAS	CASMUA	CASWUA
		IC_50_ (µg/mL)		IC_50_ (µg/mL)	
**Breast cancer**	T47D	>125	>125	>125	>125
	MCF7	>125	>125	>125	>125
	MDA-MB231	>125	>125	>125	>125
	MDA-MB468	>125	>125	>125	>125
**Prostate cancer**	PC-3	>125	>125	>125	>125
	DU145	120.01	115.86	124.87	120.97
	LNCaP	>125	69.13	>125	62.35
**Melanoma**	A375	64.53	>125	76.13	>125
	G361	123.42	>125	110.27	>125
	SK-MEL28	>125	>125	>125	>125
**Glioma**	LN229	120.68	104.25	123.21	112.69
**Gastric cancer**	AGS	>125	>125	>125	>125
**Lung cancer**	NCI-H1563	102.06	116.98	117.79	121.95
**Fibroblast**	BJ	>125	>125	>125	>125

ASE: accelerated solvent extraction; UAE: ultrasound-assisted extraction; CASMUA: *Centaurea castriferrei* Borbás & Waisb ultrasound-assisted extraction methanol–water (7:3 *v*/*v*) extract; CASWUA: *Centaurea castriferrei* Borbás & Waisb ultrasound-assisted extraction water extract; CASMAS: *Centaurea castriferrei* Borbás & Waisb accelerated solvent extraction methanol–water (7:3 *v*/*v*) extract; CASWAS: *Centaurea castriferrei* Borbás & Waisb accelerated solvent extraction water extract; T47D, MCF-7, MDA-MB231, MDA-MB468: human breast cancer cell lines; PC-3, DU145, LNCaP: human prostate cancer cell lines; A375, G361, SK-MEL28: human malignant melanoma cell lines; LN229: human glioblastoma cell line; AGS: human gastric adenocarcinoma cell line; NCI-H1563: human non-small cell lung cancer cell line; BJ: human normal fibroblast cell line.

## Data Availability

The data presented in this study are available on request from the corresponding author.
